# Co-infection associated with SARS-CoV-2 and their management

**DOI:** 10.2144/fsoa-2022-0011

**Published:** 2023-02-03

**Authors:** Vivek P Chavda, Aayushi B Patel, Anjali Pandya, Lalitkumar K Vora, Vandana Patravale, Zara M Tambuwala, Alaa AA Aljabali, Ángel Serrano-Aroca, Vijay Mishra, Murtaza M Tambuwala

**Affiliations:** 1 Department of Pharmaceutics & Pharmaceutical Technology, L M College of Pharmacy, Ahmedabad, Gujarat, 380009, India; 2 Pharmacy Section, LM College of Pharmacy, Ahmedabad, Gujarat, 380058, India; 3 Department of Pharmaceutical Sciences & Technology, Institute of Chemical Technology, Mumbai, 400 019, India; 4 School of Pharmacy, Queen's University Belfast, 97 Lisburn Road, BT9 7BL, UK; 5 College of Science, University of Lincoln, Brayford Campus, Lincoln, LN6 7TS, UK; 6 Department of Pharmaceutics & Pharmaceutical Technology, Yarmouk University, Faculty of Pharmacy, Irbid, 566, Jordan; 7 Biomaterials & Bioengineering Lab, Centro de Investigación Traslacional San Alberto Magno, Universidad Católica de Valencia San Vicente Mártir, c/Guillem de Castro 94, Valencia, 46001, Spain; 8 School of Pharmaceutical Sciences, Lovely Professional University, Phagwara, 144411, India; 9 Lincoln Medical School University of Lincoln, Brayford Campus, Lincoln, LN6 7TS, UK

**Keywords:** antiphospholipid syndrome, chronic pulmonary disease, co-infections, COVID-19 complications, COVID-19 life cycle, happy hypoxia, immuno-oncological challenges, MIS-C, mucormycosis, risk factors, SARS-CoV-2, systemic disorders

## Abstract

SARS-CoV-2 was discovered in Wuhan, China and quickly spread throughout the world. This deadly virus moved from person to person, resulting in severe pneumonia, fever, chills and hypoxia. Patients are still experiencing problems after recovering from COVID-19. This review covers COVID-19 and associated issues following recovery from COVID-19, as well as multiorgan damage risk factors and treatment techniques. Several unusual illnesses, including mucormycosis, white fungus infection, happy hypoxia and other systemic abnormalities, have been reported in recovered individuals. In children, multisystem inflammatory syndrome with COVID-19 (MIS-C) is identified. The reasons for this might include uncontrollable steroid usage, reduced immunity, uncontrollable diabetes mellitus and inadequate care following COVID-19 recovery.

## Plain language summary

COVID-19 infection has reported in the development several other infections and co-morbidity in patients. The present review discusses risk and management strategies in patients suffeting from co-infections caused by COVID-19 infection.

Coronavirus is a diverse group of viruses that are frequently and rapidly passed on from individual to individual via respiratory droplets when an individual comes into proximity to that coughing and sneezing ferociously [1,2]. The rate of secondary transmission of the novel coronavirus increases with an increase in contacts. When the virus enters the human body, the incubation period is 5–6 days to 14 days [3]. During the presymptomatic stage, an infected person might be contagious and spread the virus to a healthy person [4,5]. Symptoms rapidly progress from moderate to severe. In certain situations, 75–80% of infected people reported moderate signs, presumably a fever, tiredness, cough, cold, and loss of taste and/or smell. Several individuals had infrequent symptoms such as headaches, sore throats and skin rashes [6]. Over 40% of patients had asymptomatic COVID-19, and almost 15% of patients were critically sick and needed emergency oxygen supply. COVID-19 symptoms might differ depending on the intensity of the infection and with different variants of SARS-CoV-2 [7]. The most current form of COVID-19 (Omicron) is also infecting quite a lot of people. It exhibits symptoms comparable to hypoxia and shortness of breath [8]. Detection of COVID-19 is done by real-time (RT)-PCR techniques, which include estimation of the nasopharyngeal and oropharyngeal swab. This test is repeated for the confirmation of viral clearance in COVID-19 patients [9–11].

As COVID-19 is a respiratory tract infection, Lungs are mostly affected, and manifestations may differ from mere asymptomatic or mild pneumonia to severe pneumonia, hypoxia, respiratory failure, multiorgan failure and even mortality [12–16]. In addition, lymphopenia (hematological complication) and neurological manifestations are reported [17]. We attempted to elucidate the prevalence of delayed and long-term symptoms in COVID-19 patients, as well as to define COVID-19 associated complications and secondary infections. In addition, we have discussed majority of the post-covid associated co-infections and their care. Numerous organs that SARS-CoV-2 has damaged have been described. The purpose of this study is to offer all available information on post-covid complications and their treatment.

## Mutations, recombination & emerging variants of SARS-CoV-2

Recently, many mutated variants of SARS-CoV-2 surfaced all across the globe [18]. Initially, the Alpha variant was found in the UK in September 2020. Similarly, A Beta variant was identified in South Africa, while a Gamma variant was reported in Japan and Brazil in late 2020 [19]. A more lethal variant, in other words, Delta variant and Delta plus variants, were identified in India in 2021, which is spreading rapidly and marked their presence in 114 countries [20–24]. SARS-CoV-2 mutations have been classified as variations of interest (VOI), variants of concern (VOC), and variants of high consequence by the WHO. At the present, four VOCs and several VOIs have been identified and require ongoing monitoring [25–27]. Omicron was discovered in Botswana in November 2021 and was designated as a VOC by WHO. It is critical to developing a proven vaccination approach that is effective against the majority of VOCs [21,28,29]. The mutation of these variants continues to evolve in individuals [30]. To conquer this global pandemic; huge immunization campaigns are needed worldwide [31]. Following the Alpha, Beta, Gamma and Delta outbreaks, the most frequently emerging variant of concern (VOC) is Omicron (B.1.1.529). Omicron has several mutations that lead to increased transmissibility and evasion from immunity acquired after infection or via vaccine [32–35]. Viruses endure genetic modifications and develop new varieties by two fundamental mechanisms: the first is the random occurrence of point mutations, and the second is recombination, in which RNA viruses exchange huge segments of genetic material [36]. Earlier, recombination was seen within the viral lineages of MERS-CoV, the coronavirus responsible for the Middle East Respiratory Syndrome. The UK Health security agency validated the recombinant variants of SARS-CoV-2. This health agency also confirmed two more SARS-CoV-2 recombinant variants, XE and XF, in addition to recombinant variant, ‘Deltacron’ (XD). The XD variation incorporates genetic components from the Delta and Omicron BA.1 variants [28,37].

By changing the neutralizing activity of antibodies and monoclonal antibodies, the VOCs have a powerful effect on the COVID-19 vaccines, leading to a mild-to-substantial reduction of effectiveness [28,38,39]. Currently, 129 vaccines are under clinical trials, and we have 38 approved vaccines that are efficient in opposition to different variants of SARS-CoV-2 [11,22,40–43]. As of July 2022, there have been more than 12 billion vaccine doses have been delivered globally, and over 61.9% global population is fully vaccinated. Even though there is a disparity in vaccination efforts in various nations, every effort is being made to manage and prevent this infection [44]. VOCs have been linked to increased transmissibility or virulence, decreased neutralization by antibodies obtained from natural infection or vaccination, the capacity to elude detection and a reduction in therapeutic or vaccine efficiency [45]. To protect against variants that result in lowered vaccination effectiveness, booster vaccines have been implemented in many countries [46,47]. Furthermore, Moderna intends to test three booster methods, including a variant-specific candidate, a multivalent candidate, and a third dose booster candidate [48]. However, providing booster doses may diminish the vaccines' potential interchangeability. Booster doses are often given as a series of injections from the same vaccine manufacturer [49].

## Current status of treatment for SARS-CoV-2

No specific drugs are found to completely treat COVID-19, and several drugs are repurposed to check the efficacy against COVID-19. More than 600 clinical trials are ongoing for different therapeutic. Primarily corticosteroids are used for 3–5 days to manage lung inflammation, although it manifests a deterioration in oxygen saturation [50]. Research studies suggest that methylprednisolone with a dose range of 0.555–1.0 mg/kg/day can reduce mortality of moderate COVID-19 cases [1,51,52]. Recently, preliminary low-dose dexamethasone therapy has been effective in acute instances of COVID-19 [53]. Remdesivir at first proposed the therapy of COVID-19 under emergency use approval by the US FDA. It works by interfering with the function of viral RNA-dependent RNA polymerase (RdRp) [54]. Lopinavir that is approved for HIV is also evaluated along with many anti-HIV drugs against SARS-CoV-2 [1,55]. Similarly, Favipiravir manifests inhibitory action to RdRp [56,57]. Nirmatrelvir is an orally accessible protease inhibitor that inhibits MPRO, a viral protease that cleaves the two viral polyproteins and is required for viral replication. Nirmatrelvir is combined with ritonavir (as Paxlovid), a potent CYP 3A4 inhibitor. Here, Ritonavir is necessary to raise nirmatrelvir concentrations to therapeutic levels [58]. Molnupiravir is a prodrug of beta-D-N4-hydroxycytidine (NHC), a ribonucleoside with antiviral action against RNA viruses. When NHC is taken up by viral RNA-dependent RNA-polymerases, it causes viral mutations and fatal mutagenesis. Molnupiravir is an effective antiviral agent against SARS-CoV-2. Molnupiravir, as a mutagenic ribonucleoside antiviral drug, has the danger of being metabolized by the human host cell and integrated into the host DNA, which will lead to mutation [58,59]. The immunomodulatory drug tocilizumab is also suggested for treating COVID conditions to manage the cytokine storm condition [60].

Multiple SARS-CoV-2 lineages have been identified since its emergence, with a few classified as VOCs due to their impact on public health. The commercial sale of bamlanivimab plus etesevimab and casirivimab plus imdevimab has been suspended in USA due to the fact that the Omicron version has dramatically decreased *in vitro* sensitivity to these maAbs. Bebtelovimab continues to be effective against the SARS-COV-2 Omicron strain, as well as BA.1.1.529, BA.1.1 and BA.2. In the BLAZE-4 research study, bebtelovimab offered equivalent protection to a combination of monoclonal antibodies [61]. These results demonstrate that emerging variants of SARS-CoV-2 has not only impacted vaccination efforts but also to the therapeutic management.

## COVID-19 & post COVID-19 associated complications

While there are similarities between long COVID and post-SARS and post-MERS syndromes, the clinical and biochemical symptoms coupled with prolonged COVID seem to encompass many organ systems. SARS-CoV-2 eventually affects multiple organs, resulting in severe organ damage [62]. COVID-19 mainly affects the lung, heart, brain, kidney, liver and GI tract. Therefore, precautions should be taken, and consultations with patients when discharged from the hospital should be provided to understand other possible abnormalities that may occur in the post-covid recovery phase [63]. Among the many factors, one of the factors is an increase in the rates of blood clots observed in COVID-19 patients, which will generate a lot of downstream issues [64]. A better comprehension of the sustained consequences of the disorder will help practitioners and authorities to develop guidelines for conclusively supporting patients who survived COVID19, given that the long-term consequences of COVID19 are anticipated to place a physical, mental, and financial burden on patients, caregivers, and healthcare systems (Table 1).

**Table 1.** Estimated data of post-COVID-19 complications.

Countries	Cases of post COVID-19 complications				Ref.
	Mucormycosis	Candidiasis	MIS-C	Happy hypoxia	
India	>40,854	>9000	>3000	>1500	[65–68]
USA	>100	–	–	–	[66,69]
UK	>500	–	–	–	[66,70]
Mexico	–	–	–	>500	[71]
Japan	–	–	–	>100	[72]
Canada	>300	–	–	–	[65]
Australia	>100	–	–	–	[73]

### Methodology

**Figure 1.**
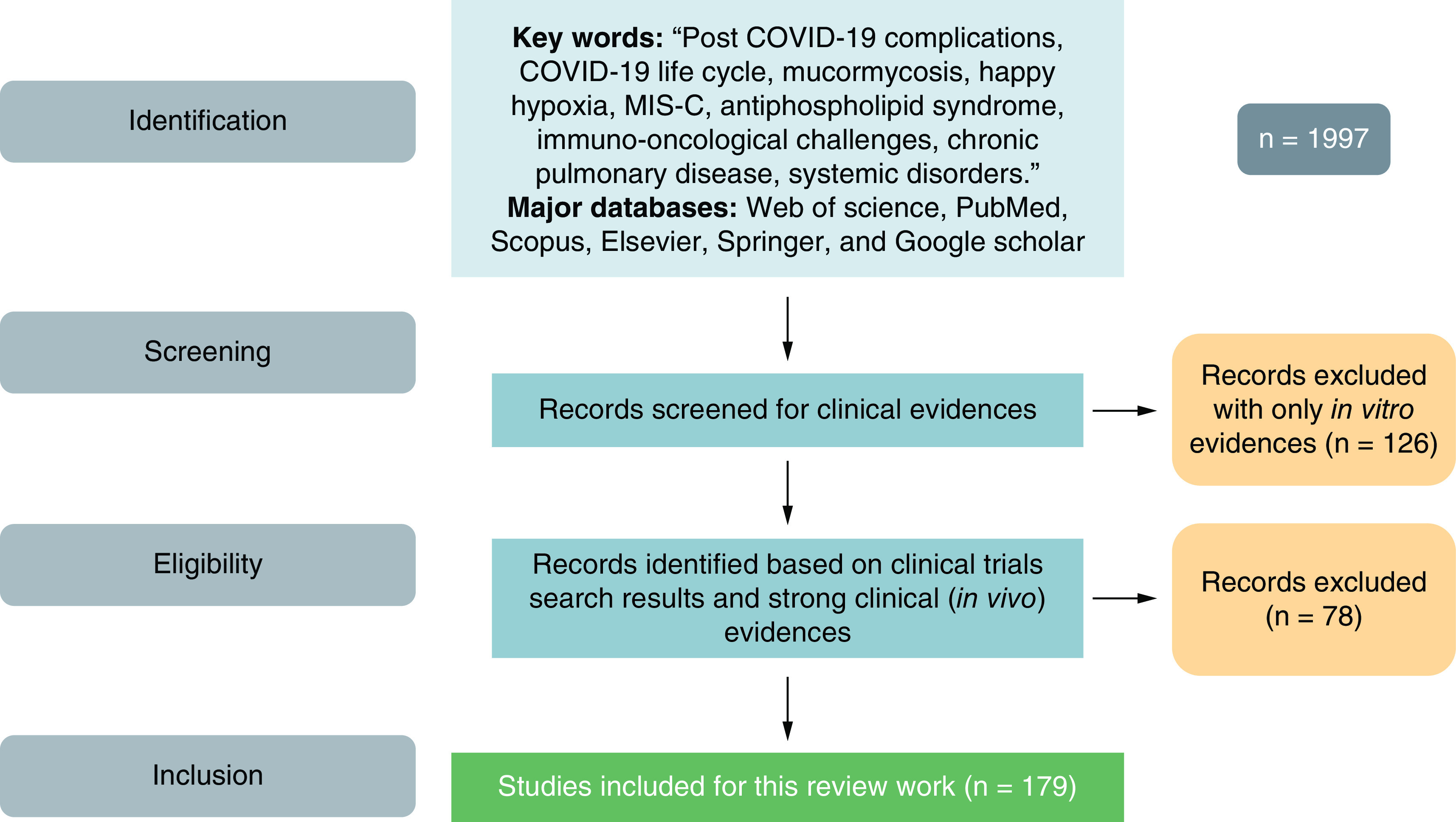
Methodology for inclusion and exclusion of the work.

We have searched through major databases like Scifinder, Web of Science, Scopus and PubMed with key words like, ‘Post COVID-19 Complications’, ‘Systemic disorders with COVID’, ‘Organ-specific sequelae with COVID’, ‘Secondary infections with COVID’, ‘post-COVID syndrome’, ‘Long COVID’, ‘COVID-19 associated complications’, etc. Figure 1 summarizes the methodology used for searching of the literature. Duplicates and non-english articles were excluded from the study alog with articles for which we do not have full texts.

### Organ damage due to SARS-CoV-2 infection

In most cases, mild or severe COVID-19 symptoms persist around two weeks. In others, however, the long-term consequences of COVID-19 may cause persistent health issues and months of devastation [74]. The pathogenesis of SARS-CoV-2 contains numerous variables that lead to severe damage, first in the lungs and subsequently via systemic spread to other organs. In addition to a broad range of hematological abnormalities and neurological diseases, multi-organ dysfunction is characterized by acute lung failure, acute liver failure, acute kidney damage, and cardiovascular disease [50,75]. Even though the existence of angiotensin-converting enzyme 2 was verified in the lung, heart, kidney, liver, and nervous system, there are some concerns regarding the presence of SARS-CoV-2 RNA in these organs [62]. More study, patience, and health education are necessary to comprehend and identify the post COVID problems in a variety of demographics and contexts, which will potentially help us to comprehend the infection's long-term implications. The convergence of slow acute inflammatory response, autoimmune and viral latency may lead to the pathophysiology of post-COVID-19 disorders. First, lets understand the organ damage by long COVID.

#### Lungs

COVID-19 can produce extended damage to the small branches of the trachea in the lungs (alveoli). Scar tissue in the lung can cause long-term breathing problems [76]. Pneumonia linked to COVID-19 can cause dysfunction in the lungs [62]. SARS-CoV-2 damages the immune system, and the cytokine storm causes lung failure, and other organ damage that can cause death in severe cases [77]. Several pathways of pulmonary fibrosis in COVID-19 have been described, including viral and immune-mediated processes. Aside from these, other factors may increase susceptibility to severe lung injury, increasing the risk of fatalities or pulmonary fibrosis in the covid recovered patients [78–80]. While there was no identifiable explanation for post-COVID-19 lung fibrosis, there seem to be a number of risk variables, including advanced age, smoking habits, a high CT severity score, and long-term mechanical ventilation. By using early medical care measures, such as anti-fibrotic medications, illness morbidity and death rates may be decreased [81].

#### Heart

**Figure 2.**
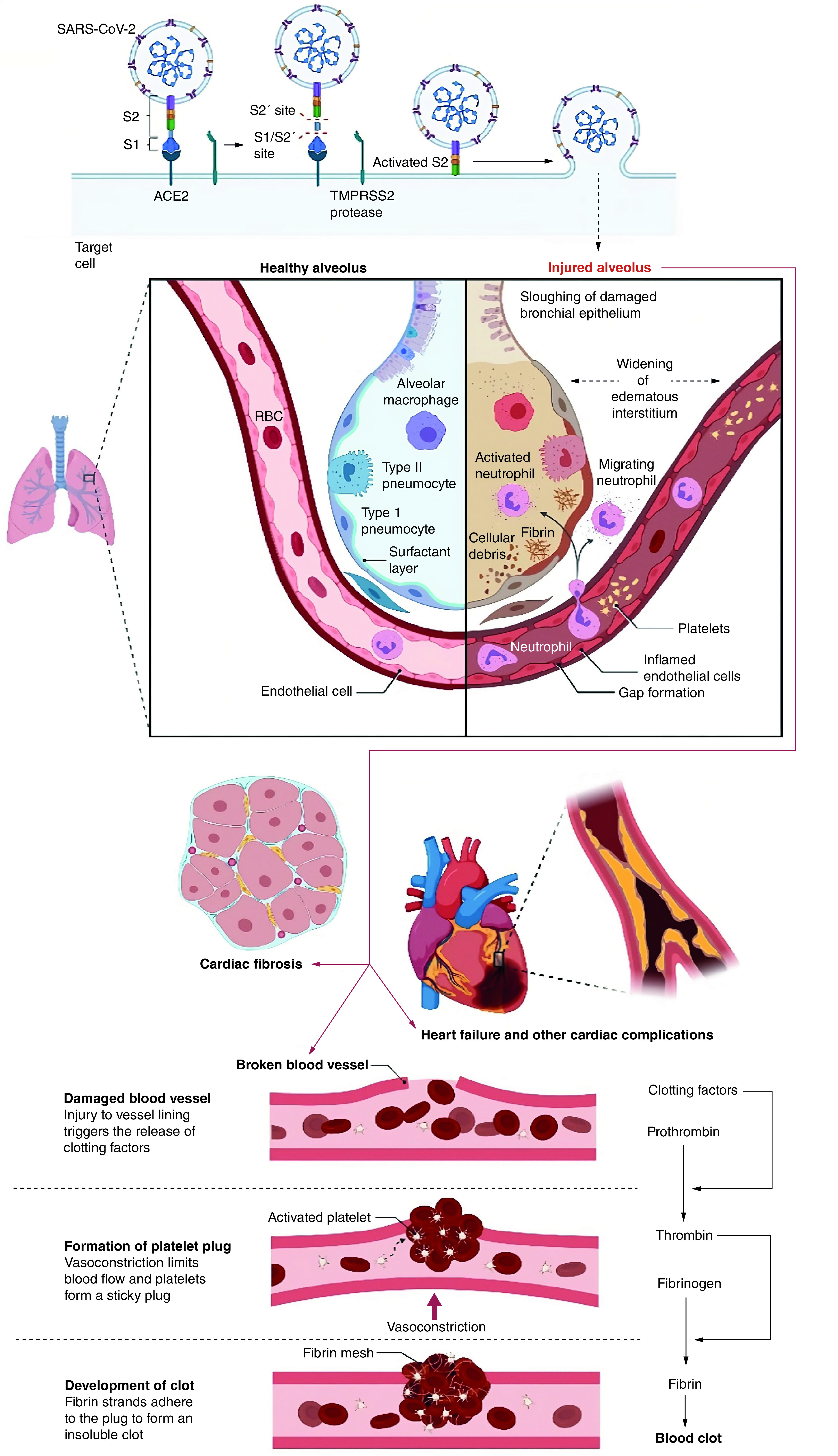
Mechanistic representation of cardiovascular complications that may occur during the post-COVID-19 recovery phase. Figure was originally created with biorender.com

Several things can damage the mechanism of the heart. As per the reports, SARS-CoV-2 can create severe myocardial inflammation, and low synchronization of ACE2 will lead to cardiac failure or myocardial abnormalities in patients [40]. The discharge of elevated production of cytokines as a major aspect of the inflammatory reaction in severe COVID-19 can harm numerous tissues, including vascular endothelium and cardiac myocytes [82]. Individuals who suffered hypoxia as a consequence of acute respiratory distress syndrome (ARDS) and also cytokine storm from systemic inflammation which can destruct cardiac muscle tissue [83]. Recovered patients of COVID-19 have shown acute heart failure, chronic dilated cardiomyopathy, myocardial infarction, cardiac arrhythmias, and irreversible tissue death of the heart muscle or other heart-related life-threatening complications (Figure 2) [84,85]. Cardiomyopathy, a heart muscle illness that inhibits the heart's capacity to pump blood adequately, may be caused by viral infections. When the body is under assault by a virus, it experiences stress and generates catecholamines, which may cause the heart to stop [86]. Treatment and early identification of cardiovascular disease using clinical and laboratory indicators are of the utmost significance. Clinical studies are now ongoing, and more research will be conducted to examine the illness and its medicines, which will assist doctors in treatment and policymakers in establishing recommendations for the improved management of COVID-19′s cardiovascular components.

#### Brain

In recuperated patients, transmembrane protease serine 2 (TMPRSS2), and ACE2 are diagnosed relatively less in the brain cortex [87]. The substantia nigra and brain ventricles are highly expressed with ACE2, and its expressions are detected in neurons comprising excitatory and inhibitory neurons, with some non-neuron cells such as oligodendrocytes, and astrocytes [88]. Many reports have shown that younger patients of COVID-19 are also experienced neurological symptoms including muscle weakness, tingling, or numbness in the hand and feet, which can eventually cause paralysis (Guillain-Barre syndrome), dizziness, sudden blackout, confusion, delirium, seizures, stroke, and may also raise the likelihood of getting Alzheimer's and Parkinson's disease [63,89,90].

Neuropsychiatric diseases, such as mental disorders, can induce brain damage or sickness as a direct result of CNS infection or as a result of an immune response or therapy [91]. Viral infection of the brain can lead to a variety of neurological and mental issues, resulting in a severe stage of the disease and its prognostic implications. The overall immune response contributes to the pathogenesis of many neuropsychiatric disorders [92]. Changes in cognitive functioning will be a key clinical starting point, which is being expedited by pandemic developments in digital health. Premorbid brain health may considerably optimize risk variables, and established therapeutic frameworks give valuable direction for addressing COVID-19 nerve systems that are overstressed [93].

#### Kidney

COVID-19 may lead to renal cell injury and can also develop acute kidney injury (AKI). AKI commonly worsens the course of COVID-19 hospitalizations and is linked to higher disease severity, longer hospitalization length, and poor prognosis. Given the severity of AKI's deleterious effects, early diagnosis of comorbidities and renal problems is crucial for enhancing the prognosis of COVID-19 patients [94]. Tubular injury is suggested by low molecular weight proteinuria, Fanconi syndrome, and histological findings. Crumbling glomerulopathy has been found in individuals with severe APOL1 genetic variants, most of whom do not have chronic lung health problems [95]. Provincial inflammation, endothelial injury, and renal microthrombi have been confirmed, but their role in the pathophysiology of COVID-19-related AKI has not been established [96]. Due to virus infection, cytokine storm and direct cellular infection through ACE2 can lead to renal damage [97]. Hypoxia, hemodynamic instability, rhabdomyolysis, and shock can indirectly cause kidney damage [62]. Chronic renal impairment may need dialysis or other treatments after COVID-19 recovery.

#### Liver

Virus invasion directly gives rise to frequent bile acid accumulation in COVID-19 patients [98]. It infects the liver cells, causing liver and cholangiocyte damage. In diagnostic tests, plasma concentrations of markers of liver function such as total bilirubin, gamma-glutamyl transferase, and transaminase were significantly higher in individuals with severe COVID-19 infection [99]. A direct influence, drug-induced damage, and hypoxia result in acute liver failure [100]. The catastrophe of COVID-19 has been associated with enhanced alcohol consumption, an unhealthy diet, and disruptions in hepatology services, which may increase the occurrence and progression of liver disease [98]. In summary, increased liver enzymes are often moderate and typically resolve without therapy in COVID-19 illness. In clinical practice, we must determine if aberrant liver function begins at the time of diagnosis or during therapy. In addition, a patient with severe COVID-19 is a complicated individual who may be exposed to pharmacological polypharmacy, which increases the risk of liver damage. The effects of COVID-19 infection and anti-COVID-19 therapy on liver function need a comprehensive study and more high-quality studies [101].

#### GI tract (GIT)

Post-COVID recovered patients may primarily suffer from diarrhea, nausea, vomiting, and abdominal pain. In GIT, the stomach, ileum, colon, and esophagus show high ACE2 in absorptive intestinal epithelial cells (IECs). ACE2 and TMPRSS2 show higher co-expression and extreme binding abilities of SARS-CoV-2 for ACE2 and its located on the mature enterocytes in the colon and ileum [102]. All these activate the TMPRSS2 and TMPRSS4, leading to epithelial cell fusion connecting viral invasion of enterocytes of the digestive tract, and it damages the GI tract [100].

## Complications associated with COVID-19

Most of the COVID-19 patients who recover expeditiously or even recovered completely have a mild symptomatic condition. Although convalesced from COVID-19 persist to experience few symptoms even after four weeks after initial recovery, they are exhibiting post-COVID complications [103]. Like, Mucormycosis is a rare condition induced by contact with a class of moulds known as micromycetes [104]. COVID-19 patients who have unmanaged diabetes and weakened immune systems are more likely to acquire this disease. The use of steroids as a therapy for the severely and critically ill covid patient is another main reason for the fast spread of mucormycosis [70]. Many recovered COVID-19 patients are having hypercoagulation disorders, varying from disseminated intravascular coagulation to venous thromboembolism, all of which can contribute to cerebrovascular disease [105]. Antiphospholipid antibodies and the lupus anticoagulant (LA) are signs and symptoms of antiphospholipid syndrome (APS), which also includes venous and arterial thrombosis [106].

Cardiovascular complications may occur in the post-COVID recovery phase, leading to a cardiac arrest and, in severe cases, a heart attack [86]. COVID-19 is an inflammatory disease that produces inflammation in blood vessels also. An increase in the thickness of blood also appears that leads to clot formation in the lungs and heart arteries causing symptoms like shortness of breath, very fast or slow heart rate, dizziness and unconsciousness in individuals who are in the process of healing from COVID-19 [107]. Apprehensions have also been increased about the neuropsychiatric consequences of SARS-CoV-2 infection. For months after discharge, COVID-19 survivors may experience recurring malaise, diffuse myalgia, cognitive impairment, depression and anxiety [108]. Gastrointestinal symptoms in SARS-CoV-2 patients arise when the infection is co-related along the lung–intestine–brain axis, where the virus triggers intestinal receptors, increases tissue inflammation, and leads to a high viral load [109]. In epidemiological investigations of COVID-19 patients, diarrhea, acid reflux, vomiting, stomach discomfort, anorexia, gastrointestinal bleeding, loss of appetite and constipation were reported. Renal problems are prevalent in people who have been treated for COVID-19 [110]. AKI is lethal in COVID-19 patients [107,111]. Children have also been hospitalized with multiple COVID-19-related symptoms, such as MIS-C, fever, stomach discomfort, vomiting, diarrhea, rash, headache and elevated inflammatory markers that may occur in children with post-COVID-19 infection, as well as neurological and cardiovascular problems [112]. Maculopapular rashes, urticaria, vesicles, petechiae, purpura, chilblains, lived or racemose and distal limb ischemia is some dermatological problems associated with COVID-19 [113].

### Immune dysfunction

Patients with COVID-19 have an abnormal immunological response. This syndrome is characterized by a reduction in T cells, B cells and NK cells as well as an increase in inflammatory cytokines [114]. In extreme cases, they were also discovered to have elevated levels of white blood cells, neutrophils and D-dimer. Furthermore, the severe group had decreased lymphocyte, CD4^+^ T cell, CD8^+^ T cell, NK cell, and B cell counts [115]. According to a multivariate logistic regression study, CD4^+^ cell count, neutrophil-to-lymphocyte ratio (NLR), and D-dimer were possible causes for extreme situations. The severity of the illness was linked to both the CT score and the clinical pulmonary infection score (CPIS). All of these factors showed a strong predictive value, according to the receiver operating characteristic (ROC) curve study [116]. Immune dysfunction is important in disease progression. For early, screening of severe cases, early and consistent surveillance of total blood cell count, T lymphocyte subsets, coagulation function, CT scan, and CPIS was indicated [117]. The dysregulation of the aforementioned immunological markers, as well as bacterial coinfection, were major causes of the patients' condition worsening and mortality [115,116]. As per antiviral immune reaction against the virus, antibody-based immunotherapies of COVID-19 include injection of convalescent plasma from recovered patients, high-dose intravenous immunoglobulins (IVIG), monoclonal antibodies, and polyclonal antibodies [118]. Also, cell-based treatment, vaccine-based approaches, cytokine-based immunotherapy, immune checkpoint inhibitors, JAK inhibitors, decoy receptors, and immunosuppressive drugs are used for the management of such immune conditions [119,120].

### Mucormycosis

As the COVID-19 global pandemic is evolving, extra impediments linked with COVID-19 are emerging [121]. Among the diversity of hindrances stated throughout and post COVID-19 infection, there has been a substantial rise in occasional occurrences of paranasal sinus mucormycosis in COVID-19 patients [121,122].

The interrelation between coronavirus and mucormycosis has been noticed through the use of copious components. Treatment of SARS-CoV-2, especially in severe cases, necessitates critical treatment requiring ventilator support and administration of corticosteroids that seize the accompanying massive airway inflammation [104,123]. Unmanageable diabetes mellitus (DM) and other immunosuppressive conditions make a more significant hazard of mucormycosis in someone vulnerable. The period allying with the detection of COVID-19 and the evolution of indications of mucormycosis is likely 15.6 ± 9.6 days [124,125].

Originally known as zygomycosis is generated by a fungus of the order Mucorales such as *Mucor, Cunninghamella, Absidia, Rhizomucor* and *Rhizopus* linked with human infections and owning 11 genera and 27 species [70,126]. In recent years, an alteration in the epidemiology of mucormycosis has been scrutinized by the latest activators and susceptible populations related to elevated morbidity and mortality rate [126,127]. Mucormycosis is infrequent; however, it is an acute invasive fungal infection predominantly seen primarily in immunocompromised patients with issues of orbital and cerebral inclusion in diabetic ketoacidosis and subsequent avail onset of steroids, as well as neutropenia, organ transplantation, and elevated levels of free iron [127–129]. Cellular immune dysfunction arises since severe pneumonia destructs alveolar epithelial and endothelial tissues [126]. Hematologic malignancies (HM), acute neutropenia, disorderly diabetes mellitus, deferoxamine treatment, solid organ transplant recipients (SOTRs), extended corticosteroids application, stem cell transplantation, iron overload, prohibited intravenous drug use, neonatal prematurity, malnourishment, and inherent nosocomial roots (intravascular implements as well as bandages) are the frequent hazardous features for the mucormycosis [126,130]. Nasal biopsy and ensuing culture are the abundant modes of discovering mucor [130]. Global rise in mucormycosis instances connected with hematologic neoplasm and organ transplant beneficiaries [131]. Mucorales attains a prone host by inhalation, consuming fouled meals, or abraded skin, resulting in rhino–orbito–cerebral, pulmonary, gastrointestinal, or cutaneous/wound infections, respectively [132]. Angioinvasive action of mucormycosis out-turn into vascular thromboses and eventually tissue necrosis [133]. Mucormycosis is influenced by ketoacidosis and deferoxamine, the significance of hyperglycemia, iron, and acidifying ketone molecules in Mucorales cynicism has been discovered [125,129,134]. As per clinical and experimental statistics, individuals with phagocyte insufficiency or weekend phagocytic function are in soaring jeopardy of mucormycosis, for instance, patients with AIDS and acute neutropenic patients [127]. The research shows that neutrophils, but not necessarily T lymphocytes, are required for preventing fungal spore growth. Furthermore, synthesizing oxidative metabolites, cationic peptides, and defensins kills two of the normal ost's mononuclear and polymorphonuclear phagocytes [128,134].

The practice of concurrent immunomodulatory drugs like tocilizumab could raise the prospects of infections in COVID-19 patients [70,135]. In India, Diabetes Mellitus was noted in 54–76% of instances of mucormycosis, and almost 8–22% had diabetic ketoacidosis. Non-uniform check-ups in Indian citizenry result in detecting mucormycosis unmasked diabetes in 43% of sufferers from North India, 24% in South India, and 40% in Western India [129,136]. In Europe, the USA, and Australia, hematological malignancies (HM) and hematopoietic stem cell transplantation (HSCT) are general intrinsic defects in mucormycosis about 40-49%. Whereas, in India, a risk factor is 1–9% [129,130]. 2.6–11% of mucormycosis manifestations were reported by SOTRs. Due to immunocompetent patients, the risk factor is 3–26%. Chronic kidney disease (CKD) is recent jeopardy for mucormycosis in India. The instances of mucormycosis with CKD is 9–32%. In comparison, pulmonary tuberculosis and Chronic Obstructive Pulmonary Disease (COPD) were spotted in 7–46% of mucormycosis patients [137]. To achieve recuperation, recognition requisites rapidness and a high index of inkling owing to the violent continuation [131]. Diagnosis of suspected mucormycosis called for physical assessment, and comprehensive history is essential [128]. In India, current guidelines suggest methylprednisolone 0.5–1 mg/kg/day for three days in mild cases and 1–2 mg/kg/day in severe cases [138,139]. The National Institute of Health (NIH) recommends dexamethasone (6 mg per day for a maximum of 10 days) in ventilated patients. Aside from that, the recommendations hint at the possibility of a secondary infection developing [134,138]. COVID-19′s pathophysiologic properties may allow for secondary fungal infections. Immune dysregulation caused by COVID-19, including fewer T lymphocytes, CD4^+^T cells, and CD8^+^T cells, may alter innate immunity [122,124,134,138]. Amphotericin-B deoxycholate with its liposomal formulations is a good place to start because of its lower nephrotoxicity. However, surgical debridement of the afflicted region must be performed as soon as the prognosis has been verified. Although simple surgery is ineffective, an adversarial surgical strategy has improved survival [104,140]. The first-line recommended antifungal medicines are liposomal amphotericin (L-Amb) or amphotericin-B lipid complex (ABLC) [132,141].

### Candidiasis

Along with increasing instances of mucormycosis infections among COVID patients, many cases of severe candidiasis infections have lately been reported in India [142]. It is a fungal infection that originated via yeast called *Candida albicans* [143]. This fungus usually exists on the skin and in the body's core, like the throat, mouth, gut, and vagina. It can cause infections if it raises uncontrollably [142,144]. The sores attack the esophagus and give rise to struggle in swallowing food, and in the mouth, white patches are found [142,143]. Lack of immunity or exposure to the things that hold these molds, like water, etc., can be the reason for the infection [144]. Lungs and other various parts of the body, including the kidney, brain, stomach, mouth, nails and vagina, are affected mainly by White fungi [145]. Appropriate sanitation can reduce conditions, and CT scans or x-rays are used to diagnose disease conditions. It can be cured by antifungal medication [146]. The three main classes of antifungals prescribed for the treatment of candidiasis are azoles, polyenes, and echinocandins [147]. *Candida spp.* isolates were found in patients that were submitted to: tocilizumab, tocilizumab plus systemic steroids, interferon type 1β and Lopinavir-Ritonavir [148].

### Happy hypoxia

**Figure 3.**
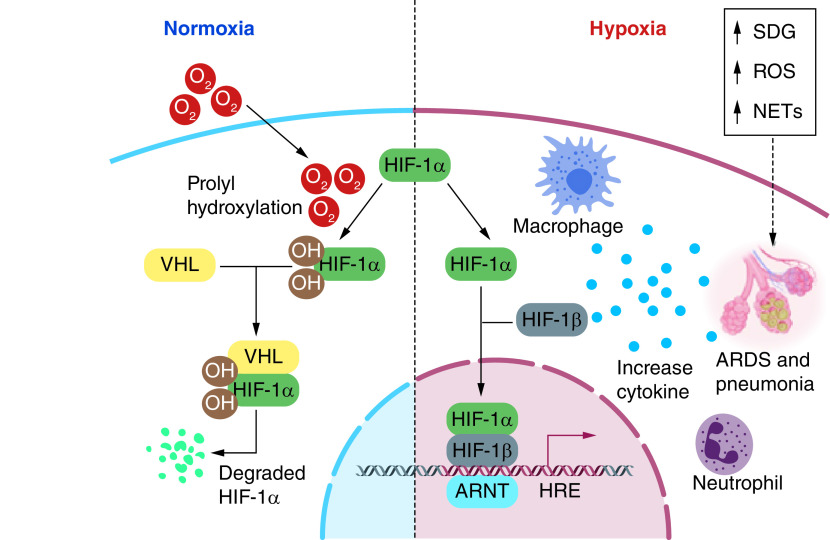
SARS-CoV-2-induced pneumonia causes chronic hypoxemic respiratory failure that is attributed to acute respiratory distress syndrome. Figure was originally created with biorender.com ARDS: Acute respiratory distress syndrome.

COVID-19 pneumonia creates chronic hypoxemic respiratory failure attributed to ARDS (Figure 3) [149]. Term hypoxemia is elucidated as a reduction in the partial pressure of oxygen in the blood. A person may encounter shortness of breath (dyspnea) as blood oxygen levels decrease [150]. Happy hypoxia is also named ‘silent hypoxemia’, which is the phenomenon that results because of the presence of hypocapnia (decrease in alveolar and blood carbon dioxide levels) [151]. SARS-CoV-2 persuade vascular proliferation in the lungs exhibited both in anatomic and radiologic studies. Patient with COVID-19 lacking radiologic lung lesions has a late right-to-left intrapulmonary shunt via contrast increased echocardiography. This right-to-left shunt will convince hypoxia [152]. In COVID-19 infected patients, coagulation in the multiplex network of small blood vessels in the lungs is the initial cause of happy hypoxia [72,149]. Patients with lessen oxygen saturations (SpO_2_ <90%) and patients with severe COVID-19 infections are required to have ICU (intensive care unit), which have high CER (case fatality rate) can suffer from happy hypoxia [71,150,153]. According to Negri and Collegues [154], “*PaO2/FiO2 ratio increased significantly over the 72 h following the start of anticoagulation, from 254(± 90) to 325(± 80), p = 0.013, and 92% of the patients were discharged home within a median time of 11 days. There were no bleeding complications or fatal events.*” For adults with COVID-19 and acute hypoxemic respiratory failure despite conventional oxygen therapy, the experts recommends starting therapy with HFNC oxygen; if patients fail to respond, NIV or intubation and mechanical ventilation should be initiated (BIIa).

### Multisystem inflammatory syndrome in children with COVID-19 (MIS-C)

In children, SARS-CoV-2 infection is often mild and asymptomatic [155,156]. MIS-C is unusually connected with COVID-19, imparting infection as elevated inflammation, high fever and organ disruption 4–6 weeks later [155,157]. Following an initial wave of COVID-19 hospitalization, MIS-C is emerging in youngsters, frequently bringing vascular involvement and shock [158]. MIS-C has clinical symptoms similar to Kawasaki illness, and it is thought to be a post or delayed condition [157,158]. CD4^+^ and CD8^+^ T-cells were noted greater in pediatric patients and fell off with age. Consequently, subsequent T-cell analysis is conditional to the prevalence of the non-native subset [158]. SARS-CoV-2 has demonstrated the ability to cause an increase in the state of post-infectious myocarditis, and fundamental pathophysiology is found to be parallel across every age [112,157].

CDC circulated a health advisory demonstrating statistics and facts regarding a receivable case of MIS-C. An individual aged <21 years can be affected along with clinical criteria of at least 24 hours of fever ≥38.0°C, more than one organ dysfunction [159]. Cardiac inclusion is persistent in MIS-C, which leads to an extensive state of affairs, likely acute myocardial dysfunction or arrhythmias, systemic hyperinflammatory conditions, and severe supplementary conditions named cardiogenic shock or hypotension [160]. A study of MIS-C patients found that intravenous immunoglobulin (IVIG) or tocilizumab (TCZ) administration was probably effective, except for P3 (priority three casualties), for whom IVIG was kept [161,162].

### Antiphospholipid syndrome

**Figure 4.**
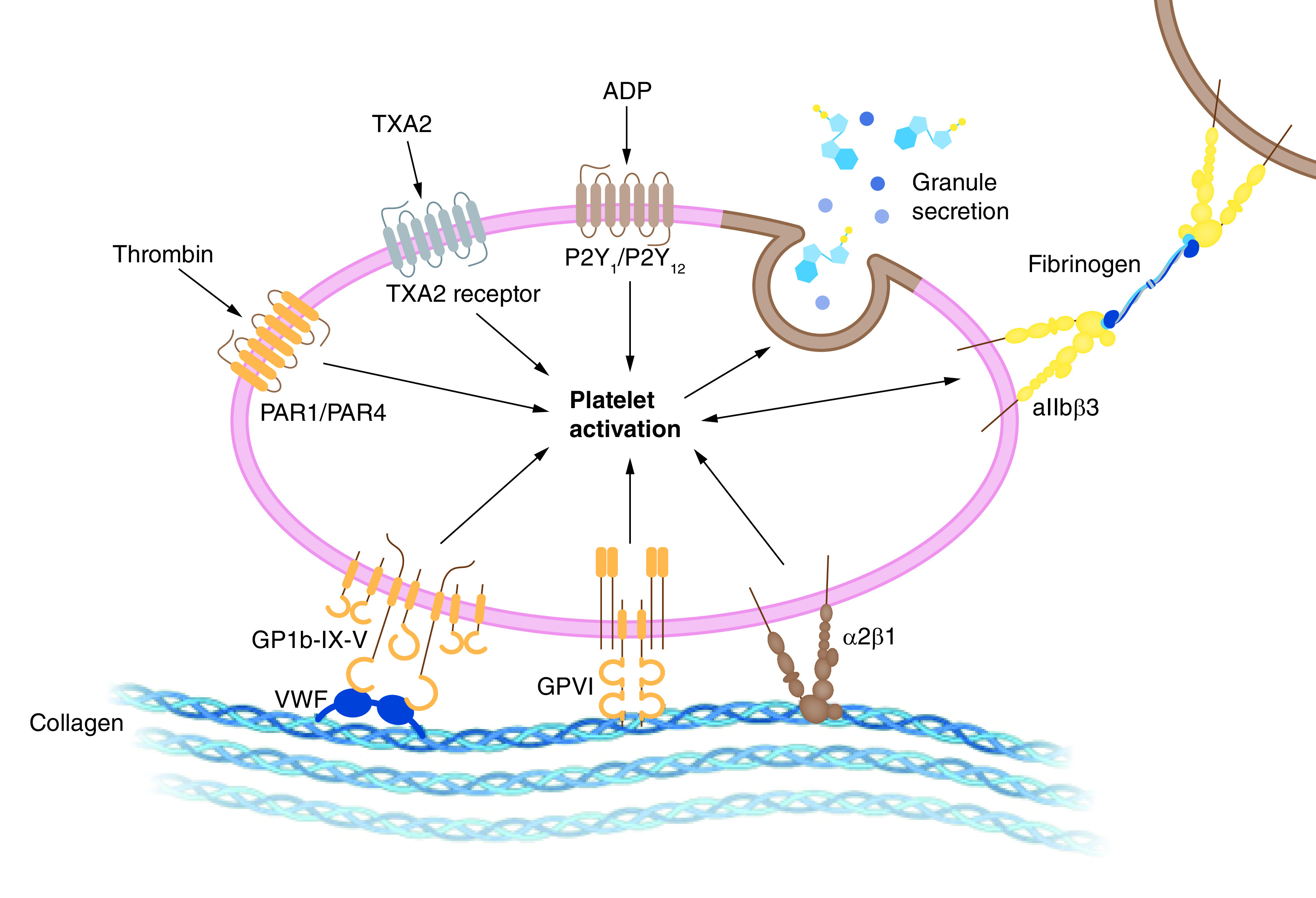
A mechanistic overview of SARS-CoV-2-induced platelet activation that leads to secondary complications such as antiphospholipid syndrome and blood clots. Figure was originally created with biorender.com

APS is an autoimmune prothrombotic or thrombophilia disease. APS occurs when the immune system generates inaccurately elevated antiphospholipid antibodies (aPLs), also known as autoantibodies, that assemble blood much more likely to abnormal thrombosis, block the blood flow, and damage the major blood organ in the body [163]. Recently, numerous clinical studies developed that show multiple emerging documented reports associated with COVID-19 infection with a higher-than-expected frequency of amended coagulation parameters. Half of the COVID-19 patients under medical treatment have intense hypercoagulable circumstances [164]. Most venous and arterial thrombosis instances occur in the lower limbs and cerebral artery circulation, respectively. Antiphospholipid antibodies can result in disability, significant sickness, and major pregnancy problems such as eclampsia, preeclampsia, stillbirth, miscarriage, as well as other difficulties such as thrombocytopenia [165]. Additionally, original evidence proposes that COVID-19 patients having a higher frequency of aPLs and major clots are seen in a small blood vessel in the lung called microthrombi. Furthermore, scintigraphic interpretation of pulmonary vessel of COVID-19 patients established ubiquitous thrombosis with capillary wall become so thick and fragile that lead to bleeding occurrence and leak the protein and slow down the blood flow [166]. The positive APS COVID-19 patient with old age and co-morbidities-related complications, including high blood pressure, hypercholesterolemia, hyperglycemia, and inflammatory disease such as rheumatism, can create severe problems in patients [167]. People with ARDS can be seen in severely sick COVID-19 individuals and have an inappropriate degree of exertion, and multiorgan dysfunction has been illuminated as a major etiology of morbidity in COVID-19 [13,168]. Antiphospholipid antibodies are commonly linked with the interrelation of viral infections such as coronavirus, human immunodeficiency virus (HIV), hepatitis B virus (HBV), Epstein–Barr virus (EBV), and parvovirus B19 (aPLs) [164]. The factual facts and written reports resulted from careful pathological investigation, such as thrombolysis [165,169]. If the COVID-19 patient has some toxic habits such as tobacco, smoking, alcoholism, and metabolic syndrome can be a conversation of natural immune defense phospholipid antibodies into active antiphospholipid antibodies that cause the development of thrombosis in patients [164]. However, clinical evidence and epidemiological study find out that the risk of cardiovascular disease and stroke can develop the APS and lupus antibodies increased in those women who smoke and continue taking oral estrogen pills [170,171]. In COVID-19 patients, bacterial and viral coinfections such as Borrelia Burgdorfer, EBV, HIV, leptospira, and treponema have been linked to developing aPLs [172]. Certain medications can interfere in APS progression, including chlorpromazine, heparin, anti-seizure drug phenytoin, procainamide, heart rhythm regulating quinidine, and hydralazine. If COVID-19 patients face another autoimmune disorder such as lupus or Sjogren's syndrome, it would be generated aPLs. A mechanism behind flaring/triggering of autoimmunity disorders associated with COVID-19 has been suggested [173]. Autopsy reports of COVID-19 patients specify that the disease may cause another obstacle due to inflammation and thrombophilia, including transient ischemic attack, red rashes, continuous bleeding episodes, swelling, and infertility [174]. In rare cases, SARS-CoV-2 vaccines can cause severe thrombotic events [175]. Antiphospholipid antibodies are a diverse collection of autoantibodies directed against an epitope on a plasma protein that binds to anionic phospholipids on the plasma membrane and is associated with thrombophilia [170]. The pathological investigation discovered that lupus anticoagulant (LA) positive is prevalent in pediatric patients who were hospitalized for SARS-COV-2 infection. Anticardiolipin antibodies (ACL) IgG and IgM, Anti beta 2 glycoprotein 1 antibodies (Anti2GP1) IgG and IgM, and LA antibodies are the three recognized aPLs [176]. Anti2GP1 is a complement control protein that is the primary target of aPLs [60]. The trimeric transmembrane S glycoprotein comprises two subunits: the S1 subunit is important for virus attachment to the host cell receptor ACE2 on endothelial cells, and the S2 subunit aids in viral capsid fusion with the host cell membrane [177]. Once it attaches to the receptor, the S protein is cleaved by a host protease, allowing the virus to enter the host cell via endocytosis. After SARS-COV-2 is ingested in a host cell, the virus releases the single-stranded RNA to the cytoplasm, hijacks the cellular machinery, and produces systemic and vascular inflammation. This inflammation is caused by releasing fibrinogen, IL-6, CRP, F VIII, and VGF, which are associated with hypercoagulability (Figure 4) [164]. The immunogenic process that is then triggered includes a network of numerous pro-inflammatory factors such as toll-like receptor 4 (TLR-4) generating a cytokine storm, which is accompanied by endothelial alterations that result in a procoagulant state [178–180]. Endothelial damage might cause second hits, causing redox equilibrium to be disrupted. The disturbance of redox equilibrium caused by monocyte and neutrophil generation of reactive oxygen species resulting in reduced antioxidant capacity leads to pro-inflammatory and prothrombic states in a patient with APS [181]. Latest upgrades to clinical outcome statistics from COVID-19 participants indicated the medication containing enoxaparin, tinzaparin, and heparin as an anticoagulant to decrease mortality [182]. Nonetheless, recent research found heparin resistance in certain individuals with COVID-19 infection, as defined with the need for a dosage of unfractionated heparin more than 35,000 IU/day to achieve the desired aPTT ratio. Glucocorticoids are anti-inflammatory medications that are widely used in APS, such as Alteplase, Tocilizumab, Methylprednisolone, Dexamethasone, Prednisone, and Hydrocortisone. Triple medication with glucocorticoids, anticoagulants, and plasma exchange with or without IVIG was linked with a reduced death rate in CAPS than no therapy [180]. However, a medical trial involving a person with severe COVID-19 who had effective antiphospholipid antibodies implement plasmapheresis and intravenous immunoglobulin (IVIG) that averting mechanical ventilation, indicated that eradication of aPLs and inflammatory cytokine stabilizes the endothelial membrane and re-establishment coagulation pathway with the use of fresh frozen plasma as a volume replacement. Complement cascade inhibitors, The humanized monoclonal antibody such as Eculizumab, Anti-C5 mAb is used to treat unmanageable CAPS by inhibiting the cleavage of C5 into C5a and C5b and reducing the chemoattractant function [166]. Hydroxychloroquine inhibits disruption of annexin A5 by Anti beta 2 glycoprotein 1 antibodies and reduces TLR-7 activation *in vitro*, reducing thrombosis in APS mouse models [171,178]. Antioxidant therapy including, N-acetyl cysteine, statin, and co-enzyme Q10, is used as adjunctive therapy for APS based on a few clinical data observed in animal model studies [180].

### Chronic pulmonary disease & COVID-19 (asthma & lung cancer)

Patients with comorbidities appear to be more common to be diagnosed with SARS-CoV-2 and at the chance of expanding acute respiratory failure. According to estimates, 10-20% of the worldwide population suffers from asthma or chronic obstructive lung disease (COPD) [183,184]. Asthma is a typical respiratory illness separated by persistent airway inflammation, increased mucus production, hyperresponsiveness, and remodeling. As a result, patients with asthma and COPD are possibly more vulnerable to critical COVID-19 outcomes, as upper and lower airways are infected with viral infections, which are significant causes of hospitalizations and exacerbations [185]. It has been discovered that type 2 immune responses, which include type 2 macrophages, T helper (Th) 2 cells, IL-4-secreting nature killer (NK) and natural killer T (NKT) cells, group 2 innate lymphoid cells, type 2 B cells, mast cells, eosinophils, and basophils, are primarily responsible for asthma [186]. IL-4 and IL-3 play key roles in allergen-specific immunoglobulin (Ig) E production, Th2 cell and eosinophil accumulation in local tissues, and epithelial barrier control, whereas IL-5, IL-9, and IL-13 boost eosinophilia and mucus formation [185,187]. Because of the increased sensitivity and severity of COVID-19 in asthmatic patients, there should be a reduced antiviral immune response and an increased likelihood of viral-elicited aggravation. SARS-CoV-2 makes use of the ACE2 receptor [188–190]. However, it was believed that asthmatic individuals are protected against COVID-19 due to type II inflammatory response cytokines (IL-4, IL-5, and IL-13) and eosinophil build-up. As a result, increased ACE-2 expression is likely to increase sensitivity to COVID-19, whereas reduced ACE2 receptor gene expression in respiratory epithelial cells in asthma patients may be the cause of protection from COVID-19 infection [191]. Another theory relates to the anti-inflammatory action of inhaled corticosteroids (ICS) and their detrimental influence on the virus-induced cytokine storm [187,188]. Furthermore, type II inflammation is connected with raised levels of TMPRSS2 (transmembrane serine protease 2), a protease required for effective viral receptor binding, as well as gene expression [192]. Consequently, the decrease in ACE2 gene expression is thought to balance the slight rise in TMPRSS2 gene expression, rendering asthma-associated type II inflammation a safety net against COVID-19 [184]. SARS-CoV-2, like other species of coronaviruses, can cause asthma exacerbations. However, more research into the immunopathological process is needed to assess the risk of severe exacerbations in asthmatic patients. As a result of these factors, it is recommended that asthmatic patients continue to take their maintenance medicines during the pandemic [191]. Shortness of breath and cough, and other manifestations of an asthma attack can occur in patients with COVID-19 infection. Additional symptoms consist of high fever, myalgia or fatigue, expectoration, and dyspnea [193]. Mild to severe sequelae, likely sore throat, headache, nasal congestion, and arthralgia, are noted, and fever occurs in around 77 percent of cases, whereas cough occurs in 81 percent of cases, diarrhoea occurs in 8% of cases, and dyspnea occurs in 3% of cases [109].

Azithromycin is beneficial in treating people with asthma who is not adequately managed by conventional inhaler therapy. Furthermore, azithromycin significantly impacts interferon (IFN) production by respiratory cells, a cytokine connected with innate antiviral immunity, and therefore it is beneficial in lowering the probability of acute COVID-19 outcomes. Allergen immunotherapy (AIT) is a disease-modifying treatment for allergic rhinitis [186,190]. AIT therapy can have a desensitization effect and increase immune tolerance in asthmatic patients, which may be protective against cytokine storms in critical coronavirus infections [183,194].

### The immuno-oncological challenge of COVID-19 (cancer)

COVID-19 is occurred by the respiratory pathogen SARS-CoV-2, and symptoms vary from moderate respiratory tract that self-limits sickness to severe pneumonia, multiorgan damage including the lungs and cardiovascular system, and sudden death [195]. There were chances of causing inflammatory burst and lymphopenia in case of having a condition like severe COVID-19 [196]. Patients with cancer, diabetes, hypertension, cardiovascular disease, asthmatic bronchitis, and chronic inflammatory or hepatic steatosis disorders are especially vulnerable to severe infections [195]. Cancer patients have particularly adverse reactions to COVID-19. Immuno-oncology is a form of cancer therapy used to increase the body's immune system to control and remove cancer, which is also called cancer immunotherapy. Because of priority and resource constraints, the worldwide oncology community is experiencing problems in the protection of cancer sufferers in the context of the SARS-CoV-2 pandemic, which may worsen cancer prognosis [68].

The clinical characteristics of COVID-19 that were identified in cancer patients, as well as the possible influence of cancer and anticancer intervention on patients' immune responses to SARS-CoV-2. Viral infections are more likely to impact cancer patients, and care should be made to cure them, along with the physiological interrelationship between two illnesses and practical recommendations for the delivery of anticancer drugs in infected patients with coronavirus [197]. Cancer patients are at a greater risk for COVID-19 and consequences such as hospitalization, critical care, and mortality, compared with the general population, due to metabolic problems such as diabetes and hypertension, as well as systemic immunosuppression. Accelerated cellular senescence and chemotherapeutic side effects that may exacerbate coronavirus disease [196]. According to evidence, cancer patients and immunocompromised hosts are more likely to be infected with SARS-CoV-2 and acquire a severe infection of COVID-19, according to evidence [198]. As a result, oncological patients may be more susceptible to COVID-19 and have a worse outcome [199]. Aging, obesity, immune senescence (a state of weakening immune system function), inflammation (cytokine dysregulation), immunodeficiency, lymphopenia, neutrophilia, and interferon deficiency. Cancer patients using immunotherapy medicines, cancer patients taking protein kinase inhibitors, and cancer patients undergoing transplantation are in danger of SARS-CoV-2 transmission [68,196,200,201]. Active cancer therapies such as surgery, chemotherapy, and radiation may be one of the variables linked with an elevated risk of SARS-CoV-2 infection in cancer patients [195]. Cancer kind, stage, and particular treatments in this patient cohort are risk factors for severe COVID-19 [202]. It is then cleaved by the host cell serine protease TMPRSS2, a member of the type 2 transmembrane serine protease family of proteins linked to cancer and viral infections [196,203]. Cancer patients have a greater level of ACE2 activation and are thus more likely to have adverse outcomes. ACE2 expression in lung tissue is raised in individuals with lung illness; in such cases, there is an elevated risk of different cancers, the most common of which is lung cancer, followed by oesophageal cancer and breast cancer [204]. TMPRSS2 is released at both the mRNA and protein concentrations in lung cells, and androgen receptors influence its activity in healthy prostate tissue and prostate cancer [198]. The virus spreads via the digestive and respiratory mucosa, infecting specific proteins and causing cytokine storms and alterations in leukocytes and lymphocytes [203]. The reduction in lymphocyte count implies that the virus eats immune cells and inhibits immunological activities, which interrelates with increased mortality in COVID-19 cancer patients. Cancer patients who healed from COVID-19 had lessened levels of IL-2R and IL-6. It implies that the number of cytokines and lymphocytes might be a predictive indicator in cancer patients with COVID-19 [202].

Due to nutritional deterioration among cancer patients, those are frequently a manifestation of anemia and hypoproteinemia, which directly affects their immunological competence and makes them more susceptible to respiratory infections. Patients with COVID-19 lung cancer may have critical anoxia and develop more quickly because of poor baseline lung function and tolerance [205]. Tocilizumab, a genetically engineered monoclonal antibody that works as an anti-IL-6-receptor inhibitor, is used to cure COVID-19 individuals with cytokine release syndrome because it lowers COVID-19 morbidity and death, even in cancer patients [197,206].

Corticosteroids, broadly categorized immune-suppressive effects, are thought to reduce host immune-mediated lung damage due to SARS-CoV-2 infection [198]. In a major randomized controlled study, dexamethasone, a corticosteroid often used in cancer therapy, also decreases the death of COVID-19 sufferers who require breathing assistance [198]. As adjuvant or directed therapy for cancer, immunomodulators (i.e., Thalidomide, Lenalidomide, and Pomalidomide) may have different effects on COVID-19 [195]. Androgen-deprivation therapy (ADT), received by those patients with prostate cancer, had significantly lesser developed clinical outcomes such as hospitalization and oxygen supplementation, lowering the risk of severe COVID-19 [198]. Anti-CD20 antibodies are used in various hematological malignancies to achieve B cell depletion, affecting the production of humoral immunity against SARS-CoV-2 [68]. Lopinavir/Ritonavir is a protease inhibitor that has been given to treat HIV-1 infection and has shown efficacy against the SARS-CoV-2 virus in *in vitro* and clinical studies [168,207,208]. The use of these medicines in hematologic cancer patients with HSC grafts must be balanced in contrast to the greater danger of post-graft imbalance in immunosuppressive therapy, which will result in graft-versus-host disease (GVHD) [201]. Imatinib, an oral anticancer drug also inhibits viral replication in the host cell by binding that drug molecule with RBD of SARS-CoV-2 spike protein [197] is widely used to control a variety of solid tumors and renal cell carcinoma, and it is currently being utilized to treat severe or acute COVID-19 pneumonia patients [197]. However, it is important to mention that SARS-CoV-2 transmission has been linked to two instances of full recovery of conventional Hodgkin lymphoma and follicular lymphoma [61,209,210].

## Other COVID-related disorders

### Thrombotic manifestation

Various medical observations show that more than 70% of COVID-19 patients experience hematological manifestations and continuous episodes of thrombotic events [68]. The coagulopathic characteristics of severe COVID-19 infection include lengthening of prothrombin time (PT), a fourfold rise in D-dimer concentration, raised fibrinogen levels, and presence of fibrinogen degradation products (FDP) [211,212]. The existence of fibrin tendency (amount of fibrin in the body) in a small vessel, capillary, and disseminated intravascular coagulation (DIC) has been found in autopsy investigations of COVID-19 patients [11]. Diffuse platelet microthrombi and megakaryocytes involve multiple organs causing severe clinical conditions in a COVID-19 patient with comorbidities [213]. Apart from these, the hypoxia and immobility experienced by hospitalized COVID-19 patients also trigger thrombosis [214]. The condition demands an urgent need for devising an efficient anticoagulation regimen to avoid severe thrombotic events in future patients of COVID-19 [25].

### Reproductive manifestations

ACE2 is a SARS-COV-2 active ligand. This controls the fundamental functions of the reproductive systems of both sexes [215]. This SARS-COV-2 virus can enter the male reproductive system and decrease fertility by producing orchitis and scrotal pain [216]. The proof is insufficient of the presence of the COVID-19 virus in semen; however, it is a matter of concern as this can become a potential and severe route of disease transmission [217]. Pregnant women in hospitals are less vulnerable to COVID-19 than non-pregnant women of comparable age, although they have a greater chance of ICU admission [218,219]. Severe COVID-19 in pregnancy is associated with maternal age, a moderate BMI, chronic hypertension, and diabetes. Furthermore, pregnant women with COVID-19 are at a greater risk of undergoing premature delivery [219].

### Neurological manifestations

COVID-19 neurological symptoms and consequences are widespread in the central nervous system (CNS) and peripheral nervous system (PNS) and cause skeletal muscle damage. A pre-existing neurological disorder can also increase the risk of developing these neurological symptoms while infected by COVID-19 [220]. Epilepsy, ataxia, encephalitis, decreased awareness, acute hemorrhagic necrotizing encephalopathy (ANE), and headache is some examples of CNS manifestations. Skeletal injury, anosmia, dysgeusia, chemosensory dysfunction, Bell's palsy, and Guillain-Barre Syndrome are all malfunctions of the PNS (GBS) [221–223]. The neuroinvasive nature of coronaviruses has been identified as their primary characteristic. Neurotrophic viruses can reach the brain through various routes, including both direct and indirect ways. Viruses can infiltrate the CNS by disrupting the blood-brain barrier endothelial cells and the blood-CSF barrier epithelial cells in the choroid plexus [14,16].

Furthermore, the virus can enter the CNS via olfactory, pulmonary, and enteric nervous system networks through retrograde axonal transport [221]. Hypoxemia can further worsen neuronal damage. Symptoms such as insomnia, anxiety, memory impairment, depression, and confusion are observed during acute illness. Sleeping disorder, traumatic memories, fatigue, memory impairment, irritability, anxiety, insomnia, and depression are usually observed [224]. Hydroxychloroquine sulfate can cause headaches, dizziness, and extrapyramidal disorders like dystonia, dyskinesia, and tremor. Furthermore, it may interact with several antiepileptic medications and reduce the convulsive threshold. Caution is also advised when prescribing hydroxychloroquine sulfate to individuals with neuromuscular junction problems, especially when combined with aminoglycoside antibiotics [225].

### Psychiatric manifestations

Due to enforced social isolation and self-quarantine, it is expected that changes in complex behavioral patterns over a lengthy time of the COVID-19 pandemic are predicted to promote a variety of psychiatric disease symptoms [226]. As a result, persistent COVID-19-related disease processes may result in severe mental diseases such as depression, anxiety, and sleep problems. Reports indicate that the virus can cause psychosis, mania, delirium, depression, anxiety, and confusion. Neurological disorders may be related to pro-inflammatory phenomena as their underlying cause [91]. Viral infections are frequent, and certain infections disrupt the CNS, causing neuropsychiatric syndromes that affect cognition, emotion, behavior, and perception. Severe illness of various etiologies is related to subsequent psychiatric morbidity [92]. Coronavirus has also been found in the brains and CSF fluids of individuals suffering from convulsions, encephalitis, and encephalomyelitis [227]. The repercussions of neuropsychiatric illnesses, such as mental disorders, that cause brain injury or illness can develop as a direct consequence of CNS infection or as an outcome of an immune response or therapy [228]. Viral infection of the brain may cause various neurological and psychiatric complications, leading to the severe stage of the disease and its prognosis consequences. The immune response of the whole body contributes to the pathophysiology of many neuropsychiatric diseases [226,229–231].

## Conclusion

COVID-19 seems to be moderate to severe especially in the aged population, patient with comorbid conditions, and immunocompromised patients. The duration of infection is little longer in those patients and even lead to post COVID complications after recovery. In recovered patients, several rare diseases include mucormycosis, white fungus infection, happy hypoxia, and other systemic disorders (multiple organ failure, immune-oncological challenges, antiphospholipid syndrome, and thrombosis, etc.) have been observed. Even MIS-C is diagnosed in children. The reason behind such post-complications is uncontrollable use of steroids, weakened immunity, unmanageable diabetes mellitus, intensive care given to patients in severe cases, and deficient care after healing from COVID-19. Patients are still at risk for lung disease, heart disease, frailty, and mental health disorders after they have recovered. There could also be long-term consequences of adverse reactions that arise during the process of COVID-19 therapies. These health problems are likely to impose extensive medical, mental, and financial pressures on all patient populations. As a result, a comprehensive plan for preventing and managing post-COVID-19 complications is required to mitigate their clinical, economic, and public health consequences, as well as to support patients who have experienced delayed morbidity and disability as a result. In an era when now almost all COVID-19 hospitalizations are treatable, this report highlights an important and under-researched COVID-19 sequela and the relating need for preventative measures. Aside from clinical manifestations, those with COVID frequently reported poor quality of life, psychiatric problems, and workplace issues. These people may require cross-disciplinary care that includes long-term surveillance of symptoms to find possible difficulties, rehabilitation programs, psychological counseling, and social care support. To ensure effective and efficient responses to future health issues, resilient healthcare systems are required. A thorough understanding of the disease's long-term side effects will allow clinicians and authorities to develop detailed guidance to help individuals who sustained COVID-19, given that the disease's long-term sequelae are expected to impose a physical, mental, and financial burden on patients, caregivers, and medical systems. As a result, a detailed plan must be developed to diagnose and control post-COVID-19 complications, as well as to support patients who are starting to experience delayed morbidity and disability as a result of those complications. More investigation, time, and education programs are required to better understand and recognize post-COVID problems in diverse settings and contexts, which will probably someday help us understand the infection's long-term consequences.

## Future perspective

A significant number of individuals with COVID-19 have post-infectious lingering symptoms. In a fraction of these patients, symptoms may remain beyond 12 weeks after the beginning of illness and are categorized as post-COVID-19 syndrome. Patients with moderate and critical symptoms of SARS-CoV-2 show multiple organ damage and other complications after recovering from COVID-19. History of other health conditions or diseases in patients elevates the chances of critical complications. The challenges to mental health have arisen owing to the prolonged pandemic, which has caused several lockdowns and stay-at-home situations. Analyszing diagnosis of these manifestations and explanation of therapeutic, and surgical properties of COVID-19 in individuals will enhance understanding of the pathology of the disease, which will lead to better ways of treatment and decrease morbidity and mortality rates. It is necessary to understand that even if the patients recover from COVID-19, they are at risk of possessing long-lasting manifestations of different kinds. It will be imperative to observe these complications to come up with viable precautionary strategies for their management. Collect data from patients with COVID-19, both with and without long-term symptoms, and compare them with individuals who may have never been infected with SARS-CoV-2 (PASC initiative of NIH). The program will aid in the collection of broad patient cohorts, comprising children and adults from varied backgrounds, in order to guarantee that the results are applicable to the areas most severely impacted by COVID-19. Again, it will be crucial to use cutting-edge technologies to decode the molecular heterogeneity of post-COVID condition and to develop druggable targets that are specifically adapted to these deficiencies [74].

**Executive summary**

- A comprehensive evaluation was conducted to analyze the incidence of co-infections in individuals with diagnosed SARS-CoV-2 infection.
- This work intended to assess the prevalence of co-infections in COVID-19 patients.
- The probable role of engaged platelets in raising the danger of SARS-CoV-2 co-infections and superinfections are discussed, along with their immunosuppressive consequences.
- This review shows that in recovered patients, several rare diseases including mucormycosis, white fungus infection, happy hypoxia, and other systemic disorders (multiple organ failure, immune-oncological challenges, antiphospholipid syndrome, thrombosis, etc.) have been observed.
